# Lung cancer mortality of residents living near petrochemical industrial complexes: a meta-analysis

**DOI:** 10.1186/s12940-017-0309-2

**Published:** 2017-09-26

**Authors:** Cheng-Kuan Lin, Huei-Yang Hung, David C. Christiani, Francesco Forastiere, Ro-Ting Lin

**Affiliations:** 1 Department of Environmental Health, Harvard T.H. Chan School of Public Health, 665 Huntington Avenue, Building 1, Room 1401, Boston, MA 02115 USA; 2 Department of General Medicine, Kaohsiung Medical University Hospital, No. 100, Tzyou 1st Road, Kaohsiung, 807 Taiwan; 3 Department of Epidemiology, Harvard T.H. Chan School of Public Health, 665 Huntington Avenue, Building 1, Room 1401, Boston, MA 02115 USA; 4 Department of Epidemiology Lazio Regional Health Service, Via Cristoforo Colombo, 112 Rome, Italy; 5 Department of Occupational Safety and Health, College of Public Health, China Medical University, 91 Hsueh-Shih Road, Taichung, 40402 Taiwan

**Keywords:** Lung cancer, Lung neoplasm, Petrochemical, Refinery, Petroleum, Oil and gas industry, Meta-analysis

## Abstract

**Background:**

Lung cancer, as the leading cause of cancer mortality worldwide, has been linked to environmental factors, such as air pollution. Residential exposure to petrochemicals is considered a possible cause of lung cancer for the nearby population, but results are inconsistent across previous studies. Therefore, we performed a meta-analysis to estimate the pooled risk and to identify possible factors leading to the heterogeneity among studies.

**Methods:**

The standard process of selecting studies followed the Cochrane meta-analysis guideline of identification, screening, eligibility, and inclusion. We assessed the quality of selected studies using the Newcastle-Ottawa scale. Reported point estimates and 95% confidence intervals were extracted or calculated to estimate the pooled risk. Air quality standards were summarized and treated as a surrogate of exposure to air pollution in the studied countries. Funnel plots, Begg’s test and Egger’s test were conducted to diagnose publication bias. Meta-regressions were performed to identify explanatory variables of heterogeneity across studies.

**Results:**

A total of 2,017,365 people living nearby petrochemical industrial complexes (PICs) from 13 independent studied population were included in the analysis. The pooled risk of lung cancer mortality for residents living nearby PICs was 1.03-fold higher than people living in non-PIC areas (95% CI = 0.98–1.09), with a low heterogeneity among studies (*I* ^*2*^ = 25.3%). Such effect was stronger by a factor of 12.6% for the year of follow-up started 1 year earlier (*p*-value = 0.034).

**Conclusions:**

Our meta-analysis gathering current evidence suggests only a slightly higher risk of lung cancer mortality among residents living nearby PICs, albeit such association didn’t receive statistically significance. Reasons for higher risks of early residential exposure to PICs might be attributable to the lack of or less stringent air pollution regulations.

**Electronic supplementary material:**

The online version of this article (10.1186/s12940-017-0309-2) contains supplementary material, which is available to authorized users.

## Background

Lung cancer is the leading cause of cancer deaths globally [1]. The Global Burden of Disease Study estimated that 1.7 million people died from lung cancer in 2015 [1]. Although tobacco smoking acts as one of the major risk factors for the disease [2, 3], there is still a considerable fraction of lung cancer mortality that remains unexplained [4]. This is particularly noticeable in many high-income countries, which showed an apparent trend of decrease in the smoking prevalence [5]. Therefore, research in the past two decades has focused on environmental determinants of lung cancer [4, 6].

Petrochemical manufacturing industry, defined as petroleum refining (Standard Industrial Classification code [SIC] 2911) or industrial organic chemicals manufacturing (SIC 2869), involves processes that produce and potentially emit hazardous chemicals into the surrounding air, soil, and water. These petrochemical manufacturing factories are usually clustered in an industrial area together with other manufacturing processes or industry, such as steel, coking, and thermoelectric plants [7, 8], and called petrochemical industrial complexes (PIC). Several studies have detected environmental air pollutants near petrochemical manufacturing plants [9–12] and also after occasional fire accidents at petrochemical plants [13]. Long-term exposure to the poor air quality, as well as radon, chemicals, and arsenic compounds among residents living near petrochemical manufacturing complexes raised general awareness and the need to understand the possible adverse health effects among nearby residents [4, 14].

Several epidemiological studies have explored associations between the PICs and lung cancer risks of nearby people. Given high public concerns of health, the US started several investigations of suspected cancer risks for people living nearby PICs back to the 1970s [15–17]. For example, US white males living in petroleum industry counties had 1.10- to 1.17-fold higher risks of lung cancer mortality than males in other counties [17]. Subsequent studies in Italy and UK also revealed similar results with relative risks of 1.26 and 1.04, respectively, among white females [7, 8]. Fast-growing economies in Asia stimulated by the increasing demand for petrochemicals in manufacturing sectors also faced corresponding increases of lung cancer mortalities among residents nearby PICs [18]. However, several studies reported different results. For instance, Tsai and colleagues reported that male residents living in Louisiana’s Industrial Corridor had lower risks of lung cancer compared to other Louisiana citizens, even after adjusting for age [19]. Similarly, Simonsen and colleagues reported that the risk of lung cancer was not elevated significantly in accordance with the residence proximity to the industrial area [20].

Due to the inconsistent results, our study aimed to estimate lung cancer mortality risk associated with the PICs by combining cross-country data from different studies via a systematic review and meta-analysis.

## Methods

### Data source and study selection

We selected exclusively articles from PubMed, Cochrane Library, Web of Science, Science Direct, and other sources that published before July 11, 2017. We used "(*Lung cancer* OR *Lung neoplasm*) AND (*Refinery* OR *Petroleum* OR *Petrochemical* OR *Oil and Gas Industry*)" as the search term. Two researchers—HY Hung and RT Lin—selected independently articles that met the inclusion and exclusion criteria as below.

### Inclusion and exclusion criteria

The inclusion criteria were: (1) original articles that clearly defined exposure group as residents living nearby PICs; (2) original articles that clearly defined lung cancer mortality according to International Classification of Diseases (ICD); (3) original articles that reported either confidence intervals (CI), standard errors (SE), or both; and (4) original articles that were written in English and full-texts were available. The exclusion criteria were: (1) studies with subjects overlapped with other publication; (2) studies that focused on occupational exposure in petrochemical plants only; and (3) studies that reported lung cancer incidence only and lack of mortality data.

### Review process and data extraction

**Fig. 1:**
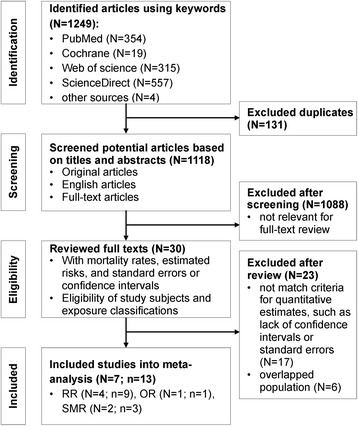
Flow of systematic literature search on lung cancer mortality for residents living nearby petrochemical sites. N = number of studies; n = number of estimates included into meta-analysis; RR = relative risk (rate ratio or risk ratio); OR = odds ratio; SMR = standardized mortality ratio

Figure 1 shows the selection process of the articles, including four steps: identification, screening, eligibility, and included. First, we identified 1249 articles from library databases and excluded 131 duplicated articles. Second, we screened articles by titles and abstracts. We chose 30 of them as relevant to our study objective for full-text review. Third, we carefully reviewed and checked whether those articles clearly defined exposure and health outcome and also reported estimates and CI or SE. Considering that a study population might appear in different articles, we selected the latest article to avoid bias towards the specific population. Finally, we included seven articles that reported 13 estimates for meta-analysis: three articles reporting sex-specific mortality rate ratios of lung cancer [7, 18, 21]; one article reporting sex-specific age-adjusted mortality rates of industrial corridors and Louisiana, respectively [19]; one article reporting odds ratios for both sexes combined [22]; one article reporting standardized mortality ratios by sex [8] and another one reporting standardized mortality ratios sex combined [23]. The ratio of Belli’s study was regarded as for males in subgroup analysis because male accounted for 84% of the study group [22].

Since lung cancer mortalities were less than 10^−3^ per year [24], we could appropriately interpret estimated odds ratios as relative risks [25, 26]. The adjusted standardized mortality ratios could be interpreted as relative risks as well because the estimates were derived from the comparison to general population in Rome [8, 27].

For the study not reporting CI or SE [19], we estimated the variances and SE of *lnRR* using following equations:

$$ Var(lnRR)= Var\left({lnR}_1+{lnR}_0\right) $$

$$ = Var\left({lnR}_1\right)+ Var\left({lnR}_0\right) $$

1 $$ ={\left(\frac{1}{R_1}\right)}^2\times Var\left({R}_1\right)+{\left(\frac{1}{R_0}\right)}^2\times Var\left({R}_0\right) $$

2 $$ SE(lnRR)=\sqrt{Var(lnRR)} $$

where Var(*lnRR*) represents the variance of natural log of relative risks (RRs); R_1_ and R_0_ represents mortality rates of the studied group and the reference group, respectively; and SE(*lnRR*) represents the standard error of natural log of relative risks.

### Statistical analysis

We applied a random-effects model to examine whether there were within- and between-study heterogeneities using the *I* ^*2*^ test [28]. We set *I* ^*2*^ less than 10% as no heterogeneity, 10%–30% as low heterogeneity, 30%–60% as moderate heterogeneity, and more than 60% as high heterogeneity based on Cochrane handbook [29]. We further did subgroup analysis by different characteristics [30], including sex, location, ethnicity, PM_10_ standard, latency period (first year of study period more than 20 years after operation year of PICs vs. less or equal to 20 years), and bona fide observation (defined as 10 or more years of observation after 20 years of PIC operations vs. less than 10 years). Then, we applied meta-regressions to investigate the possible factors of heterogeneity, including sex, ethnicity, location, year of publication, and the starting year of follow-up. We also conducted sensitivity analyses to assess the influence of individual study on the overall RR by adding one estimate into the pooled estimates gradually. Finally, we used a funnel plot and the Begg’s and Egger’s regressions for asymmetry test to examine whether there was publication bias and small-study bias. All analyses were performed using the Stata Software version 11.2 (StataCorp, TX, US). We set the statistical significance level at 0.05, using a two-sided test.

### Assessment of data quality

To assess the risk of bias in each study, the quality of each study was recorded and assessed using the Newcastle-Ottawa Quality Assessment Scale [31]. Records on data quality for each study were reviewed by CK Lin and HY Hung. We evaluated potential bias based on three categories (selection, comparability, and outcome) with eight measurements [31]. Although the discussion on the validity of the Scale remained inconclusive, the reliability of the Scale is quite fair and widely used in meta-analysis [32, 33].

### Air quality standards

Pollutants emitted from PIC might vary over time, likely due to the change of manufacturing process and pollution control technology. Since data on air quality around studied petrochemical areas were limited, we reviewed national or regional ambient air quality standards in studied countries or regions: European Union (EU), Taiwan, and the US. We summarized three air quality standards for studied countries, including total suspended particles (TSP), PM_10_, and PM_2.5_ [34–41].

## Results

Table 1 shows the basic characteristics of studies included in our meta-analysis. A total of 13 study groups were extracted, covering around 2,017,365 people living near petrochemical areas in Taiwan, Louisiana in the US, Teesside, West Glamorgan in the UK, and Brindisi, Sicily, and Rome in Italy. Seven out of 13 study groups reported RRs for males, five for females, and one for both sexes combined. The follow-up years ranged widely from 1960 to 2002. Most PICs operated at least 14 years.

**Table 1:** Basic characteristics of studies included in the meta-analysis

ID	Comparison	Study period	The started operation year of PICs	Adjusted confounders	Country	Definition of petroleum area and reference area	Study group (Number of subject)	Outcome selection	Industrial activity/ Substantial chemicals	Study design	Reference
A	Asian males, exposure group vs. reference group	1982–1991	1968 (First–fourth naphtha cracking plants)^b^	Age	Taiwan (32 counties)	Petroleum area: 16 counties with 2% or more of the population employed in petrochemical industrial complexes (PICs) Reference area: 16 matched counties with less than 2% of the population employed in PICs	Exposure group: Residents in 16 petroleum counties (*N* = 977,853) Reference group: Residents in 16 reference counties (*N* = 870,758)	Death registered in Bureau of Vital Statistics of the Taiwan Provincial Department of Health; ICD-9 code = 162	Petrochemical manufacturing/ Vinyl chloride monomer, polycyclic aromatic hydrocarbons (PAH)	Cohort study	[18]
B	Asian females, exposure group vs. reference group	1982–1991	1968 (First–fourth naphtha cracking plants)^b^	Age	Taiwan (32 counties)						
C	White males, Industrial Corridor vs. Louisiana	1990–1999	< 1970	Age	United States (Louisiana)	Petroleum area: Industrial Corridor: the industrial area of the lower Mississippi River of South Louisiana (the highest density of petrochemical facilities in the United States) Reference area: Louisiana	Exposure group: White male residents in Industrial Corridor (*N* = 186,727)^a^ Reference group: White male residents in Louisiana (*N* = 1,385,055)^a^	Death data from the University of Pittsburgh’s Mortality and Population Data System; ICD-9 code = 162	Producers of industrial and agricultural organic chemicals, plastics, synthetics, industrial inorganic chemicals/ Ammonia, methanol, phosphoric acid, nitrate compounds, formaldehyde, and PAH	Cohort study	[19]
D	White females, Industrial Corridor vs. Louisiana	1990–1999	< 1970	Age	United States (Louisiana)		Exposure group: White female residents in Industrial Corridor (*N* = 194,376)^a^ Reference group: White female residents in Louisiana (N = 1,454,083)^a^				
E	Non-white males, Industrial Corridor vs. Louisiana	1990–1999	< 1970	Age	United States (Louisiana)		Exposure group: Non-white male residents in Industrial Corridor (*N* = 98,917)^a^ Reference group: Non-white male residents in Louisiana (*N* = 646,311)^a^				
F	Non-white females, Industrial Corridor vs. Louisiana	1990–1999	< 1970	Age	United States (Louisiana)		Exposure group: Non-white female residents in Industrial Corridor (*N* = 112,079)^a^ Reference group: Non-white female residents in Louisiana (*N* = 734,504)^a^				
G	White males, Residents vs. Commuters	1960–2002	1960 (Gela plant)^c^	Age, calendar period, residence category or job category, and time since first employment	Italy (Sicily)	Petroleum area: A large petrochemical plant built in the vicinity of the town of Gela, Sicily in 1960	Males workers employed in the Gela petrochemical plant in 1960–1993 Exposure group: Residents: workers born in Gela (*N* = 1684) and in Sicilian municipalities with a probability of commuting defined by the gravity model as < 0.5 ^g^ (*N* = 709) Reference group: Commuters: workers born in Sicilian municipalities, excluding Gela with a probability of commuting defined by the gravity model as > = 0.5 ^g^ (*N* = 3234)	Data from municipalities’ registry office; ICD-9 code = 162	Oil refinery, thermoelectric power plants, producers of organic and inorganic chemicals/ Ethylene, acrylonitrile, sulfuric acid, ammonia, chlorine urea	Cohort study	[21]
H	Whites (both genders), exposure group vs. reference group	1996–1997	1961 (Brindisi petrochemical plant)^d^	Age, sex, smoking, and education	Italy (Brindisi)	Petroleum area: The petrochemical plant located in Brindisi	Exposure group: Residents in Brindisi and three neighboring municipalities(Carovigno, Torchiarolo, San Pietro Vernotico) who died from lung cancer (*N* = 95) Reference group: Random sample of residents in the same area who died from any other disease (*N* = 170)	Death register in Local Health Authority of Brindisi; ICD-9 code = 162	Petrochemical plant/ N.R.	Case- control study	[22]
I	White males, Teesside (zone A,B,C) vs. Sunderland (zone S)	1981–1991	1965 (Teesside refinery)^f^	5-year age group	United Kingdom (Teesside)	Petroleum area: Teesside (one of western Europe’s largest steel and petrochemical complexes)	Exposure group: Residents in 19 housing estates in Teesside (zones A,B,C) (*N* = 77,330) Reference group: Residents in 8 housing estates in Sunderland (zone S) (*N* = 43,485)	Death data from the former Northern Regional Health Authority	Petrochemical complex, steel complex, coking, and chemical operations/ N.R.	Cohort study	[7]
J	White females, Teesside (zone A,B,C) vs. Sunderland (zone S)	1981–1991	1965 (Teesside refinery)^f^	5-year age group	United Kingdom (Teesside)						
K	White males, residents living within 10 km circle vs. in Rome	1987–1993	1965 (Rome refinery plant)^e^	5-year age group, four levels of socioeconomic index	Italy (Rome)	Petroleum area: Circle of 10 km radius around the petrochemical refinery which began operation in the early 1960s in the area of Malagrotta, a suburb of Rome	Exposure group: Males living within a 10 km radius of the plants (*N* = 165,074) Reference group: Males in 6108 census tracts in Rome (*N* = N.R.)	Data from the geographical information mortality system; ICD-9 code = 162	Waste disposal, waste incinerators, petrochemical refinery/ Particulates, hydrogen chloride, chlorinated dibenzo-p-dioxins, dibenzofurans, PAH, chlorinated benzene, chlorinated phenols, and phthalates	Cohort study	[8]
L	White females, residents living within 10 km circle vs. in Rome	1987–1993	1965 (Rome refinery plant)^e^	5-year age group, four levels of socioeconomic index	Italy (Rome)		Exposure group: Females living within a 10 km radius of the plants (*N* = 176,315) Reference group: Females in 6108 census tracts in Rom (*N* = N.R.)				
M	White (both genders) within 3 km vs. England and Wales	1981–1991	1963 (Baglan Bay petrochemical plant)	Age, sex, index of deprivation, and region	United Kingdom (West Glamorgan)	Petroleum area: Circle of 3 km radius centered on the petrochemical plant	Exposure group: Residents living within circle of 3 km radius centered on the petrochemical plant, and death before 74 years old (*N* = 26,206) Reference group: Residents in England and Wales, and death before 74 years old (*N* = N.R.)	Death data from the Small Area Health Statistics Unit and 1981 census; ICD-9 code = 162	Baglan Bay Chemicals/ Alcohols, styrene, olefins, benzene, vinyl chloride and polyvinyl chloride	Cohort study	[23]

*N* number of subject, *N.R.* not reported, *ICD* International Classification of Diseases

^a^Source of data: 1990 Census of Population: General Population Characteristics, Louisiana. https://www2.census.gov/library/publications/decennial/1990/cp-1/cp-1-20.pdf (Accessed 10 Mar 2017)

^b^Source of data: The industrial heritage in Taiwan (In Chinese), National Science and Technology Museum, Taiwan, http://iht.nstm.gov.tw/form/index-1.asp?m=2&m1=3&m2=76&gp=21&id=7 (Accessed 13 Apr 2017)

^c^Source of data: Pasetto R, Comba P, Pirastu R. Lung cancer mortality in a cohort of workers in a petrochemical plant: occupational or residential risk? Int J Occup Environ Health. 2008;14(2):124–8

^d^Source of data: Petrolchimico di Brindisi (1969–1972) (In Italian), Tatiana Schirinzi, http://www.nove.firenze.it/petrolchimico-brindisi/ (Accessed 14 Apr 2017)

^e^Source of data: Rome Refinery, A Barrel Full, http://abarrelfull.wikidot.com/rome-refinery (Accessed 13 Apr 2017)

^f^ Source of data: Teesside Refinery, https://www.revolvy.com/main/index.php?s=Teesside%20Refinery (Accessed 19 Jul 2017)

^g^Soure of data: Signorino G, Pasetto R, Gatto E, Mucciardi M, La Rocca M, Mudu P. Gravity models to classify commuting vs. resident workers. An application to the analysis of residential risk in a contaminated area. Int J Health Geogr 2011,10:11

**Fig. 2:**
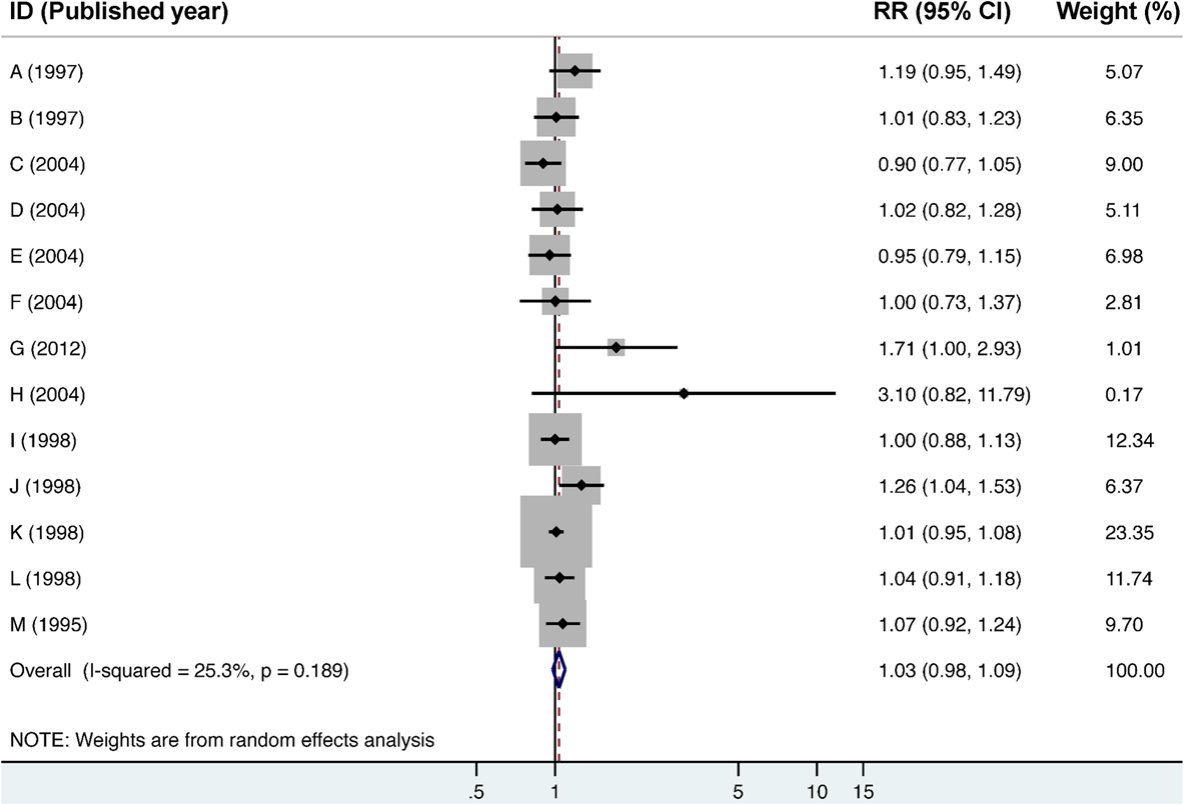
Forest plot of studies on lung cancer risks of residents living nearby petrochemical industrial complexes. RR = relative risk

Figure 2 shows the pooled estimate of mortality risk for lung cancer among residents living nearby PICs. The estimated overall RR of 1.03 indicated that lung cancer mortality among residences might be associated with exposure to PICs, but it didn’t reach statistical significance (95% CI = 0.98–1.09). Although Belli’s study (study ID = H in Fig. 2) reported point estimate of lung cancer risk as high as 3.10, its broad CI ranging from 0.82 to 11.79 led to the smallest weighting factor of 0.17% in our meta-analysis. Among the selected studies, the highest weighting factor of 23.35% (study ID = K in Fig. 2) indicated that Michelozzi’s study on males in Rome contributed to the largest proportion of the pooled estimate, mainly because this study had the narrowest CI. The overall *I* ^*2*^ was 25.3%, indicating low heterogeneity existed among these studies.

Table 2 shows the results of pooled estimates and 95% CI by different characteristics, including sex, location, ethnicity, PM_10_ standard, latency period, and bona fide observation. For each characteristic, there was no significant difference among pooled estimates between subgroups based on overlapping 95% CIs. However, we found a higher risk of lung cancer associated with residential exposure to PICs in the era of looser PM_10_ standard (RR = 1.12, 95% CI = 0.97–1.29 vs. RR = 1.01, 95% CI = 0.96–1.06).

**Table 2:** Pooled estimates of relative risks of lung cancer mortality for residents living nearby petrochemical industrial complexes, by different characteristics

Characteristics	N	Pooled RR (95%CI)	*I* ^*2*^ (%)	*p*-value
Overall	13	1.03 (0.98–1.09)	25.3	0.236
Sex				
Males	7	1.02 (0.93–1.11)	44.9	0.728
Females	5	1.07 (0.98–1.16)	0	0.137
Location				
Asia	2	1.09 (0.93–1.27)	13.6	0.311
United States	4	0.95 (0.86–1.05)	0	0.302
European Union	7	1.07 (0.98–1.16)	44.6	0.119
Ethnicity				
Asian	2	1.09 (0.93–1.27)	13.6	0.311
White	9	1.04 (0.97–1.12)	41.7	0.280
Non-white	2	0.97 (0.82–1.13)	0	0.666
PM_10_				
PM_10_ > 150 μg/m^3^	4	1.12 (0.97–1.29)	54.8	0.117
PM_10_ ≤ 150 μg/m^3^	9	1.01 (0.96–1.06)	0	0.724
Latency period				
> 20 years	9	1.00 (0.95–1.05)	25.3	0.990
≤ 20 years	4	1.10 (1.00–1.21)	35.9	0.056
bona fide observation				
≥ 10 years	6	1.01 (0.87–1.16)	39.9	0.929
< 10 years	7	1.04 (0.99–1.09)	6.8	0.115

*N* numbers of subjects included, *RR* relative risks

**Fig. 3:**
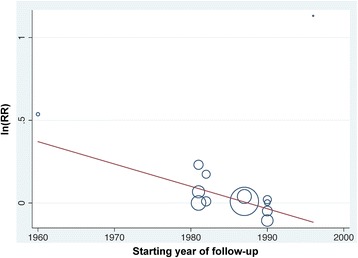
The relationship between natural log of relative risk of lung cancer mortality and starting year of follow-up. ln(RR) = natural log of relative risk

Except for the starting year of follow-up, we did not find any possible heterogeneous factor from the meta-regression analysis. The slope of the meta-regression line suggested that for an increment in the starting year of follow-up, the RR of lung cancer would be 0.874-fold lower (*p-*value = 0.034, Fig. 3).

**Fig. 4:**
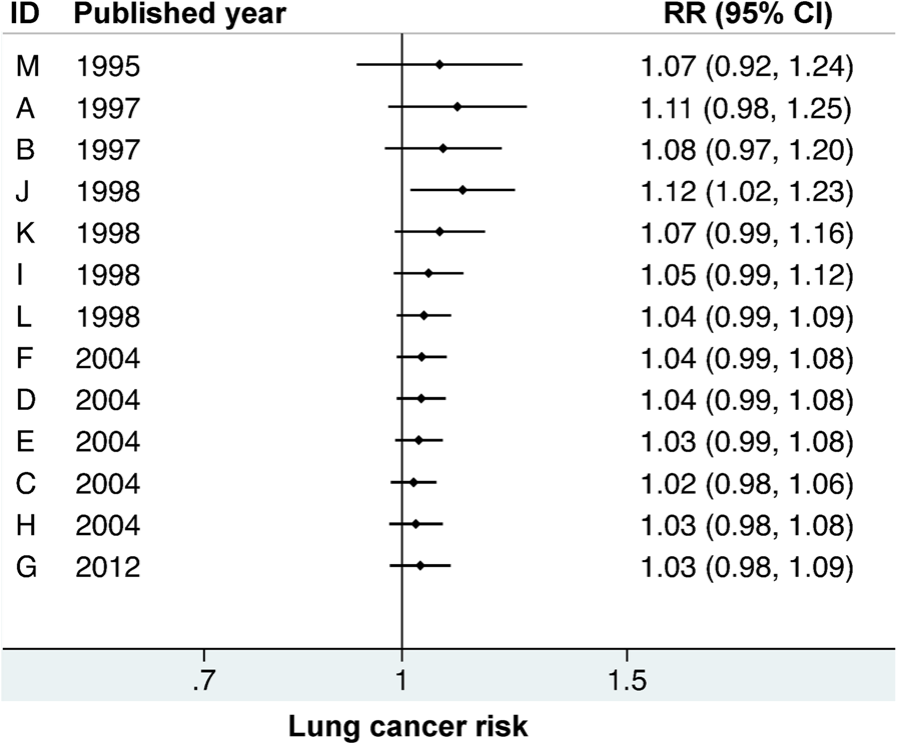
Sensitivity analysis of random effects estimates after adding each additional study according to the publication year. RR = relative risk

**Fig. 5:**
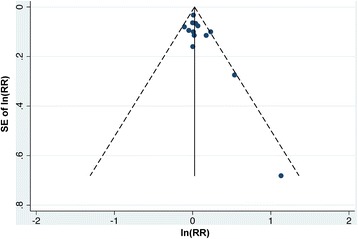
Funnel plot for lung cancer mortality relative rates associated with residential exposure to petrochemical industrial complexes of the 13 study groups. ln(RR) = Natural log of relative risks; SE of ln(RR) = standard error of natural log of relative risks

Figure 4 shows the sensitivity analysis for the effect of individual study on pooled results. We gradually added each study into the sensitivity analysis—from studies published in the earlier period to studies published in the later period. None of them significantly affected the pooled results. There was no significant publication bias among the studies for 13 study groups (Egger’s test: *p*-value = 0.059; Begg’s test: *p*-value = 0.051). The funnel plot also indicated no asymmetry for the estimates for the 13 study groups was observed (Fig. 5).

Additional file 1 listed details of the quality assessment for cohort and case-control study, respectively. All studies reported sex-specific, age-adjusted point estimates. Some studies further adjusted ethnicity, socioeconomic levels (e.g., school levels, job collars categories, unemployment, number of family members, overcrowding, and ownership of dwellings), or study periods. Four studies had full score of nine stars [18, 21–23]; two had 8 out of 9 stars [7, 8]; and one study had seven out of nine stars [19] (see Additional file 2).

**Fig. 6:**
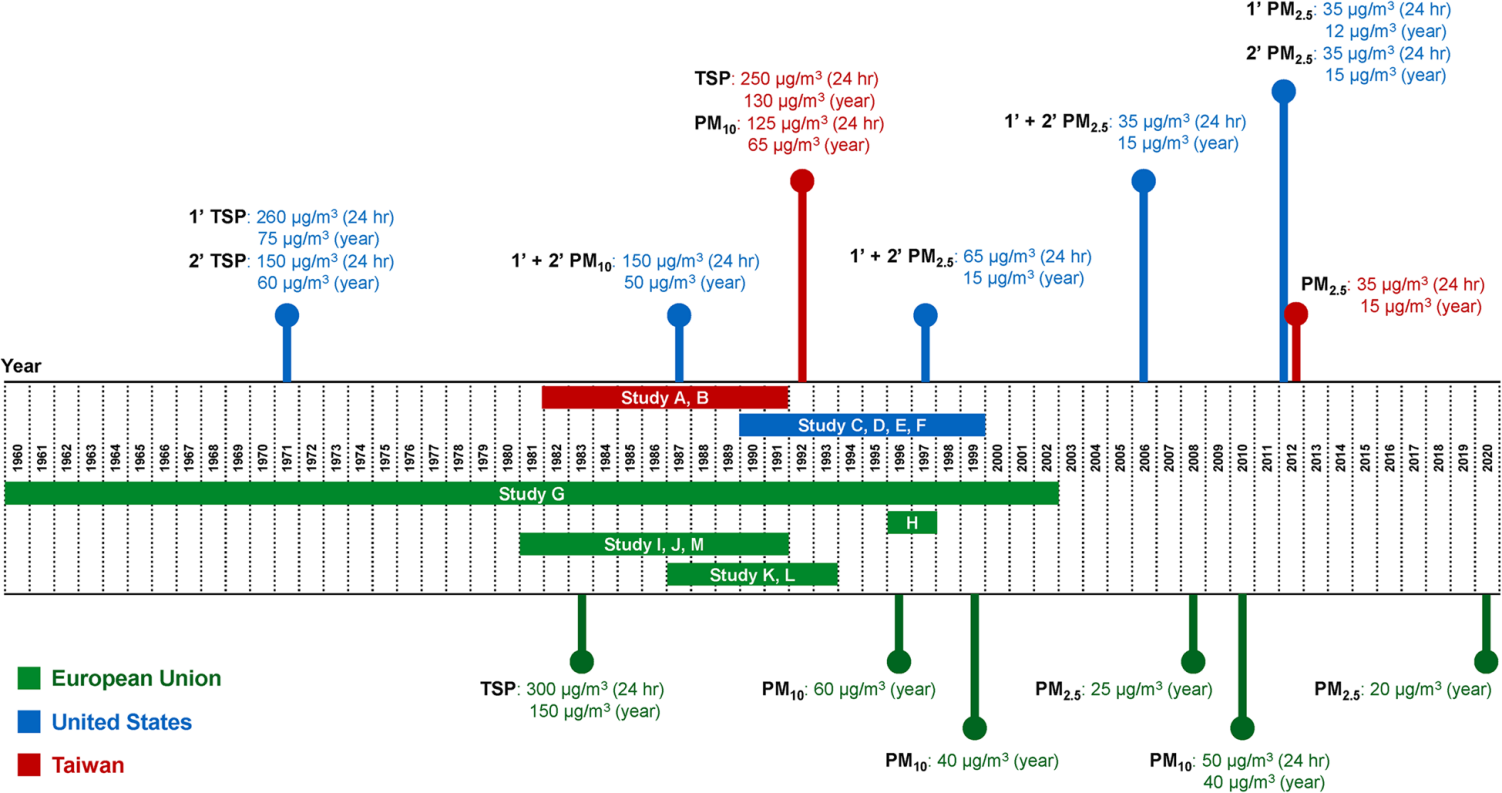
Historical air quality standards of studied regions. TSP = total suspended particles; 1’ = primary pollutant; 2’ = secondary pollutant

Air quality standards in the EU, Taiwan, and the US were summarized in Fig. 6. The earliest standard of ambient air quality was for TSP, followed by PM_10_ and PM_2.5_. All countries have set stricter air quality standards over the years. For example, the standard for annual average TSP concentration was 150 μg/m^3^ in the EU in 1983. The EU tightened the regulation by setting up annual PM_10_ standard at 60 μg/m^3^ in 1996, and then lowering it to 40 μg/m^3^ in 1999. In 2008, the EU set up the annual PM_2.5_ standard at 25 μg/m^3^. Similarly, the US set the annual TSP standard at 75 μg/m^3^ in 1971, and further tightened the limits to 50 μg/m^3^ in 1987. In contrast, Taiwan adopted the US’s 1971 standard for TSP and PM_10_ and announced the regulation in 1992, but the limits have not been changed since then.

## Discussion

To our best knowledge, this is the first meta-analysis that estimated the pooled RR of lung cancer mortality for residents living nearby PICs. We aggregated lung cancer risks for 13 study groups from seven published papers in the US, the UK, Italy, and Taiwan. Based on these studies, people living in the PICs had higher lung cancer mortality risks than residents in non-PICs by a factor of 1.03, despite such associations didn’t reach statistically significant (95% CI = 0.98–1.09). Stratification analysis by different characteristics, such as sex and ethnicity, did not change the magnitude of this association. In contrast, the starting year of follow-up affected the association between lung cancer mortality and exposure to PICs by a factor of 0.874. That is, the estimated risk of lung cancer mortality was higher among subjects recruited in earlier periods, and the risk decreased by 12.6% if the year of follow-up started 1 year later.

The scientific evidence of the study is sound and solid from several perspectives. First, the outcome variable was based on pathological samples and/or the ICD-9. Individual data were obtained by linking to governmental database. Second, the large sample size (*n* = 2,017,365) and diverse populations (e.g., by sex, ethnicities, and locations) made the pooled estimate more representative and enhanced the generalizability. Third, by applying the random-effect model, we were able to address the heterogeneity between studies and further reported the pooled effects.

We found higher lung cancer mortality risks among residents near PICs by a factor of 1.03, although this adjusted RR did not reach statistical significance. We identified the following possible limitations of the study. First, the definition of exposure varied slightly between studies. Most studies defined the exposure based on the geographical locations or distances of residencies from PIC [7, 8, 19, 21–23], while one study compared the exposed group and reference group by matching job categories in PIC and non-PIC towns [18]. Misclassification of exposure and non-exposure might exist and bias the pooled estimates towards the null. Second, the operation of PICs started as early as 1960 and some PICs are still in operation. Exposure to pollutants emitted from PICs might be quantitatively and qualitatively different in each period. Third, although our subgroup analysis didn’t show different risks for residents in different latency periods, still not everyone in the selected studies had sufficient latency periods or adequate follow-up period. The estimations on latency period for lung cancer diagnosis varied widely but usually required approximate years to decades [42, 43]. Inadequate inclusion of residents with insufficient latency might bias the result toward the null in the original studies.

An effective air quality intervention involved a series of steps, including regulatory establishments, pollution reductions, and anticipated improvements in health [44]. Although data on ambient pollution monitoring around PICs in the early periods were very limited and hard to obtain, previous studies have documented pollution reductions could be attributable to changing regulations [45, 46]. We could reasonably assume that most petrochemical factories followed the local regulations to some extent. Therefore, the historical air quality standards for TSP, PM_10_, and PM_2.5_ could reflect the relative trends of exposure to air pollutants emitted from PICs. Most air quality standards became stricter over the years [34–37, 41]. Such trend partially explains our findings in the heterogeneity regression; that is, studies on populations with earlier exposure to PICs were associated with significantly higher risk of lung cancer mortality.

There are some limitations need to be addressed when interpreting our results. First, not all potential confounders were adjusted in the seven articles, such as smoking, radon exposure, meteorological factors, and socioeconomic status. However, these unadjusted confounders posed an unknown or even lower risk of lung cancer to the exposure group compared to the reference group. For example, the smoking rate of exposure group was lower than the reference group in Bhopal and colleagues’ study [7]. Similarly, people lived in the Industrial Corridor had higher socioeconomic status (less unemployed, higher income, and higher educational attainment) compared to the average of Louisiana [19]. Since lower smoking rate and higher neighborhood socioeconomic status were associated with fewer lung cancer incidence [47, 48], health benefits from the improvement of socioeconomic status along with industrial development were likely to outweigh the negative effects of exposure to the petrochemical industry. The data on radon exposure, as one of the risk factors of lung cancer, were absent in all selected papers. However, there is no evidence of higher radon exposure in PIC areas than non-PIC ones [49, 50]. Similarly, seasonal variations of wind directions might either increase or decrease the effect of PIC exposure on residents’ health. Since all studies have exposure and reference groups from both upwind and downwind locations, subject-selection bias and meteorological effects due to location variance were reduced. Second, studies with available data for meta-analysis were originated from the US, the UK, Italy, and Taiwan. The generalization of the impact of petrochemical industry on lung cancer might be restricted to these countries. However, these four countries represented the majority of countries with the largest petrochemical industries in terms of ethylene production capacity [51], the major base of petrochemicals and a common index to estimate production capacity of a petrochemical company. Third, each PIC might involve other manufacturing processes (such as steel, cocking, and power plants) and the exposure level could also be affected by geographical factors across different countries. Limited by the lack of corresponding exposure data, our findings were not able to address the heterogeneity between PICs. Fourth, certain portion of residents living nearby PICs might risk occupational exposure as well. Some studies have separated the environmental exposure from the occupational exposure (study ID = A, B, G, H) or at least considered job categories in the analysis (study ID = M) to reduce the influence of occupational exposure.

## Conclusions

Our meta-analysis gathering current evidence suggests only a slightly higher risk of lung cancer mortality among residents living nearby PICs. Our analysis also underline the role of stringent regulations on improving air quality and reducing the residential exposure to air pollution, which can further contribute to lowering the risk of lung cancer.

## Additional files

Additional file 1: Asses﻿sment of study quality using the Newcastle-Ottawa Quality Assessment Scale for cohort and case-control studies. (DOCX 36 kb)

Additional file 2: Data quality assessment on﻿ the Newcastle-Ottawa Quality Scale. (DOCX 15 kb)

## Abbreviations

CI: Confidence interval
ICD: International Classification of Diseases
PIC: Petrochemical industrial complex
RR: Relative risk
SE: Standard error
SIC: Standard Industrial Classification code
TSP: Total suspended particles
