# Correction to: Lung cancer mortality of residents living near petrochemical industrial complexes: a meta-analysis

**DOI:** 10.1186/s12940-017-0339-9

**Published:** 2017-11-15

**Authors:** Cheng-Kuan Lin, Huei-Yang Hung, David C. Christiani, Francesco Forastiere, Ro-Ting Lin

**Affiliations:** 1 Department of Environmental Health, Harvard T.H. Chan School of Public Health, 665 Huntington Avenue, Building 1, Room 1401, Boston, MA 02115 USA; 2 Department of General Medicine, Kaohsiung Medical University Hospital, No. 100, Tzyou 1st Road, Kaohsiung, 807 Taiwan; 3 Department of Epidemiology, Harvard T.H. Chan School of Public Health, 665 Huntington Avenue, Building 1, Room 1401, Boston, MA 02115 USA; 4 Department of Epidemiology Lazio Regional Health Service, Via Cristoforo Colombo, 112 Rome, Italy; 5 Department of Occupational Safety and Health, College of Public Health, China Medical University, 91 Hsueh-Shih Road, Taichung, 40402 Taiwan

## Correction

After publication of the article [1], it has been brought to our attention that the original version of this Article contained a typo in the 3rd paragraph of the section ‘Review process and data extraction’. It concerns the equation published as “Var(lnRR) = Var(lnR1 + lnR0)”. On the right part, the “+” within the parenthesis should be “-”, as defined and derived from the left part. As a result, Var(lnRR) = Var(lnR1 + lnR0) should be revised to Var(lnRR) = Var(lnR1 - lnR0).
