# Supplementary material for: Lung cancer mortality of residents living near petrochemical industrial complexes: a meta-analysis
Source: Environ Health. 2017 Sep 26;16:101. doi: 10.1186/s12940-017-0309-2 (PMC5615452; doi:10.1186/s12940-017-0309-2)
Supplement: Supplementary file 1 — Asses﻿sment of study quality using the Newcastle-Ottawa Quality Assessment Scale for cohort and case-control studies. (DOCX 36 kb) [file 12940_2017_309_MOESM1_ESM.docx]

# Additional file 1

# Assessment of study quality using the Newcastle-Ottawa Quality Assessment Scale for Cohort Studies [1-6].

# Selection

# *Representativeness of the exposed cohort:* All studies were considered to be generally representative of the average air pollutant exposure for resident living in the community. In Yang’s study, the 16 selected petrochemical industrial counties were somewhat representative of the average air pollution situation in petrochemical industrial counties of Taiwan.

# *Selection of non-exposed cohort:* Michelozzi’s study reported the SMR, which compared the exposure group to the general population in Rome; so did Tsai’s study in Louisiana and the United States as well as Susana’s study in Wales and England. Instead of comparing to residents in Teesside, Bihopal’s study selected residences in Sunderland as the reference group. Pasetto selected “commuters” as the “non-residential exposed” group. Yang selected another 16 non-petrochemical industrial countries as compared group.

# *Ascertainment of exposure:* All studies explicitly stated how they obtained residency information. The information was used as a surrogate of exposure to air pollution in petrochemical areas.

# *Demonstration that outcome of interest was not present at start of study:* All studies got full stars on this item. The studies consistently reported mortality rates.

# Comparability

# *Comparability:* All studies controlled for age–the most important covariate in the meta-analysis. Sex, the second most important covariate, was also considered into analysis. Thus, all articles got full stars on this item. Other covariates were also controlled in the analysis, including socioeconomic index [2], job (blue or white collar) [3], calendar period [3], ethnicity [1], fraction of workers employed in non-petrochemical manufacturing industries [5], and the index of deprivation and region [6].

# Outcome

# *Assessment of outcome:* The diagnosis were obtained and verified either from pathological diagnosis or ICD coded by physicians.

# *Was follow-up long enough for outcome to occur:* All studies had at least 10 years follow-up period, which was adequate for lung cancer to occur.

# *Adequacy of follow up of cohort:* Three studies had excellent follow-up quality. Pasetto *et al.* followed at least 98.5% of patients and only about 0.7%–1.4% loss-to-follow-up in Bihopal’s study. Sans’s study had 89.3% of cancer registration and over 99% of deaths in Wales. In contrast, the other three studies didn’t state the follow-up percentage. However, we could assume that the follow-up was adequate in Yang’s study because the Bureau of Vital Statistics of the Department of Health in Taiwan has complete dataset.

# Assessment of Study Quality using Newcastle-Ottawa Quality Assessment Scale for case-control study [7]

# Selection

# *Is the case definition adequate?* Yes. The cases were ICD-9 coded in death registry in each municipality. They were further integrated by the archive of causes of death held by the local health authority.

# *Representativeness of the cases:* The cancer was well defined and representative of the cases.

# *Selection of controls & Definition of controls:* Controls were a random sample of the subjects living in the same area of exposure group and deceased in 1996–1997 for any cause except for lung cancer.

# Comparability

# *Comparability:* The results were adjusted for age, sex, smoking, and education.

# *Ascertainment of exposure:* The interviewers used a structured questionnaire and were blinded to case/control status to collect information on residential history, occupation, school level, smoking habits, and alcohol consumption of the study subjects.

# Exposure

# *Same method of ascertainment for cases and controls:* Yes.

# *Non-response rate:* 100% of response rates for the cases and 98% (6/176 refused to respond) for the controls.

# Reference

1. Tsai SP, Cardarelli KM, Wendt JK, Fraser AE. Mortality patterns among residents in Louisiana's industrial corridor, USA, 1970-99. Occup Environ Med. 2004;61(4):295-304.

2. Michelozzi P, Fusco D, Forastiere F, Ancona C, Dell'Orco V, Perucci CA. Small area study of mortality among people living near multiple sources of air pollution. Occup Environ Med. 1998;55(9):611-5.

3. Pasetto R, Zona A, Pirastu R, Cernigliaro A, Dardanoni G, Addario SP, et al. Mortality and morbidity study of petrochemical employees in a polluted site. Environ Health. 2012;11:34.

4. Bhopal RS, Moffatt S, Pless-Mulloli T, Phillimore PR, Foy C, Dunn CE, et al. Does living near a constellation of petrochemical, steel, and other industries impair health? Occup Environ Med. 1998;55(12):812-22.

5. Yang CY, Chiu HF, Chiu JF, Kao WY, Tsai SS, Lan SJ. Cancer mortality and residence near petrochemical industries in Taiwan. J Toxicol Environ Health. 1997;50(3):265-73.

6. Sans S, Elliott P, Kleinschmidt I, Shaddick G, Pattenden S, Walls P, et al. Cancer incidence and mortality near the Baglan Bay petrochemical works, South Wales. Occup Environ Med. 1995;52(4):217-24.

7. Belli S, Benedetti M, Comba P, Lagravinese D, Martucci V, Martuzzi M, et al. Case-control study on cancer risk associated to residence in the neighbourhood of a petrochemical plant. Eur J Epidemiol. 2004;19(1):49-54.
