# Supplementary material for: Lung cancer mortality of residents living near petrochemical industrial complexes: a meta-analysis
Source: Environ Health. 2017 Sep 26;16:101. doi: 10.1186/s12940-017-0309-2 (PMC5615452; doi:10.1186/s12940-017-0309-2)
Supplement: Supplementary file 2 — Data quality assessment on﻿ the Newcastle-Ottawa Quality Scale. (DOCX 15 kb) [file 12940_2017_309_MOESM2_ESM.docx]

# Additional file 2

# Table. Data quality assessment on Newcastle-Ottawa quality scale

|  | **Selection** | **Comparability** | **Outcome** |
| --- | --- | --- | --- |
| Yang *et al*., 1997 | ☆☆☆☆ | ☆☆ | ☆☆☆ |
| Tsai *et al*. 2004 | ☆☆☆ | ☆☆ | ☆☆ |
| Pasetto *et al*. 2012 | ☆☆☆☆ | ☆☆ | ☆☆☆ |
| Belli *et al*. 2004 | ☆☆☆☆ | ☆☆ | ☆☆☆ |
| Bhopal *et al*. 1998 | ☆☆☆ | ☆☆ | ☆☆☆ |
| Michelozzi *et al*. 1998 | ☆☆☆☆ | ☆☆ | ☆☆ |
| Sans *et al*. 1995 | ☆☆☆☆ | ☆☆ | ☆☆☆ |

**Reference**

Yang CY, Chiu HF, Chiu JF, Kao WY, Tsai SS, Lan SJ. Cancer mortality and residence near petrochemical industries in Taiwan. J Toxicol Environ Health. 1997;50(3):265-73.

Tsai SP, Cardarelli KM, Wendt JK, Fraser AE. Mortality patterns among residents in Louisiana's industrial corridor, USA, 1970-99. Occup Environ Med. 2004;61(4):295-304.

Pasetto R, Zona A, Pirastu R, Cernigliaro A, Dardanoni G, Addario SP, et al. Mortality and morbidity study of petrochemical employees in a polluted site. Environ Health. 2012;11:34.

Belli S, Benedetti M, Comba P, Lagravinese D, Martucci V, Martuzzi M, et al. Case-control study on cancer risk associated to residence in the neighbourhood of a petrochemical plant. Eur J Epidemiol. 2004;19(1):49-54.

Bhopal RS, Moffatt S, Pless-Mulloli T, Phillimore PR, Foy C, Dunn CE, et al. Does living near a constellation of petrochemical, steel, and other industries impair health? Occup Environ Med. 1998;55(12):812-22.

Sans S, Elliott P, Kleinschmidt I, Shaddick G, Pattenden S, Walls P, et al. Cancer incidence and mortality near the Baglan Bay petrochemical works, South Wales. Occup Environ Med. 1995;52(4):217-24.

Michelozzi P, Fusco D, Forastiere F, Ancona C, Dell'Orco V, Perucci CA. Small area study of mortality among people living near multiple sources of air pollution. Occup Environ Med. 1998;55(9):611-5.
